# CHNSO Elemental Analyses of Volatile Organic Liquids by Combined GC/MS and GC/Flame Ionisation Detection Techniques with Application to Hydrocarbon-Rich Biofuels

**DOI:** 10.3390/molecules29184346

**Published:** 2024-09-13

**Authors:** Jude Azubuike Onwudili, Morenike Ajike Peters, Carine Tondo Alves

**Affiliations:** 1 Energy and Bioproducts Research Institute, College of Engineering and Physical Sciences, Aston University, Aston Triangle, Birmingham B4 7ET, UK; petersma@aston.ac.uk (M.A.P.); carine.alves@ufrb.edu.br (C.T.A.); 2 Energy Engineering Department, Centro de Ciência e Tecnologia em Energia e Sustentabilidade, Universidade Federal do Reconcavo da Bahia, Av. Centenario 697, Feira de Santana 44085-132, Brazil

**Keywords:** volatile organic liquids, sustainable hydrocarbon-rich liquids, CHNSO, elemental analyser, GC/MS identification, GC/FID quantification

## Abstract

Elemental analysis is a fundamental method for determining the carbon, hydrogen, nitrogen, sulphur, and oxygen (CHNSO) contents in organic materials. Automated conventional elemental analysers are commonly used for CHNSO determinations, but they face challenges when analysing volatile organic liquids due to sample losses. This present study explores the combination of gas chromatography–mass spectrometry (GC/MS) and gas chromatography–flame ionisation detection (GC/FID) as a more accurate alternative method for elemental analysis of such liquids. Six different liquid samples containing various organic compounds have been analysed using both a conventional elemental analyser (Method 1) and the combined GC/MS–GC/FID method (Method 2). The results showed that Method 1 gave results with significant errors for carbon (by more than ±10 wt%) and oxygen (by up to ±30 wt%) contents due to volatile losses leading to inaccurate “oxygen-by-difference” determinations. In contrast, Method 2 gave more accurate and consistently representative elemental data in a set of simulated samples when compared to theoretical elemental data. This work proposes the use of the GC/FID method as a reliable alternative for CHNSO analysis of volatile organic liquids and suggests that employing the GC/FID technique can mitigate the common errors associated with conventional CHNSO analysis of such samples. However, successfully using Method 2 would depend on the skills and experience of users in qualitative and quantitative organic chemical analyses by gas chromatography.

## 1. Introduction

Elemental analysis of organic materials is a method that scientists use to determine the types and quantities of elements present in a material. It is often limited to the main elements expected in organic materials, including carbon (C), hydrogen (H), nitrogen (N), sulphur (S), and oxygen (O). Hence, elemental compositions of organic materials are typically reported as CHNSO. However, in most cases, the oxygen contents of such organic materials are often determined “by difference” once the CHNS contents have been accurately determined. For a wide range of solid, non-volatile, and viscous liquid samples, the ‘oxygen by difference’ estimation returns results with acceptable accuracy. However, obtaining oxygen contents “by difference” can become significantly problematic during the elemental analysis of highly volatile organic liquids and, therefore, requires more careful handling to give accurate results.

CHNSO has become one of the most important characterisations used in the scientific research of organic materials such as fuels [1,2,3], biological specimens [4,5], drugs [6,7,8], chemicals [9,10,11], biomass [12,13,14,15], plastics [16,17,18], organic wastes [19,20], and many other organic materials [21,22]. Hence, it is used in various scientific fields, including engineering, chemistry, medicine, and pharmacy, for various reasons such as chemical product development and quality control. Based on the Pregl–Dumas combustion analysis method [23], CHNSO analysis can be carried out using automated systems (CHNSO analysers) following technological developments in the last 40 years. The technique involves the reaction of organic materials with excess oxygen at high temperatures under both static and dynamic conditions. In some cases, redox catalysts such as vanadium pentoxide are added to ensure complete combustion. Essentially, the carbon, hydrogen, and sulphur atoms present are converted into carbon dioxide (CO_2_), steam (H_2_O), and sulphur dioxide (SO_2_), respectively, while nitrogen atoms form nitrogen gas (N_2_) [23]. The combustion temperature is controlled to avoid the oxidation of N atoms to nitrogen oxides. The detection of the combustion gas products can be carried out qualitatively and quantitatively using several methods. Most commonly, the combustion gases are sent into fitted gas chromatographic (GC) equipment for separation and combined detection by thermal conductivity detectors [24] or separate detection of individual gas compounds in a series of separate infrared and thermal conductivity cells [25]. Hence, these analysers can directly quantify the carbon, hydrogen, nitrogen, and sulphur contents but not the oxygen atoms in the samples.

It is important to acknowledge that direct oxygen determination can be achieved via high-temperature pyrolysis and carbonation at around 1120 °C to transform the oxygen to CO for subsequent redox reactions to quantify the oxygen [26]. This method was modified by Campanile et al., 1951 [26], to remove interferences such as hydrogen to give improved accuracy of the oxygen content determination based on iodometric titrations. Such detailed and accurate determinations of oxygen become hugely important when oxygen is the target of the analysis. Notwithstanding, for quick results, automated CHNSO analysers offer simplicity and flexibility, especially when little or no sample losses occur during the entire analytical procedure. For instance, in practice, scientists can load several samples onto the sample rack to perform multiple analyses using autosamplers and collect results later. For highly volatile organic liquid samples, losses are bound to occur during the waiting times on sample holders between analyses [27], leading to significant errors. Reducing sample waiting times and considerable losses from volatile organic samples before analysis may be minimised by soaking samples in appropriate inert absorbents [28,29] and by introducing single samples to the analyser one at a time for immediate analysis. Such practices defeat the purpose of automation, cause unnecessary delays, are unproductive, and still do not solve the problems of sample losses when analysing highly volatile organic liquids [30].

### Background

During a recent research project on the catalytic conversion of vegetable oils to liquid hydrocarbon fuels, obtaining accurate CHNSO results from the typical elemental analyser resulted in unexpected “oxygen by difference” data. Indeed, results from analyses of some hydrocarbon-rich light oil products showed significant oxygen content (by difference), even though no oxygenated compounds were identified via gas chromatography–mass spectrometry (GC/MS) analysis. Hence, it was considered that the CHNSO analyses were being affected by the volatility of the compounds in the liquid samples. Principally, the time lag between when the samples were weighed out and placed into the analyser sample holder and the actual time of analysis allowed significant amounts of volatile compounds to escape even when samples were soaked in absorbents. Unfortunately, there was no direct way of accounting for such losses given that the mass of the samples had already been recorded and entered in the processing software after weighing the samples. Hence, the software subsequently used the entered weights to calculate the CHNSO compositions, which led to significant errors.

Gas chromatography (GC) is one of the most widely used techniques for the accurate identification and quantification of volatile organic samples. Modern CHNSO analysers already have inbuilt gas chromatographic systems for separation, identification, and quantification of combustion gases [31,32]. It is an ideal technique for the analysis of gas and volatile liquid samples containing mixtures of several different organic molecules. In particular, gas chromatography fitted with mass selective detectors (GC/MS) is equipped with functionalities to enable scientists to identify the types of organic molecular species present. Thereafter, gas chromatography with flame ionisation detection (GC/FID) can be used to accurately determine their concentrations. Therefore, when used correctly, the combination of GC/MS and GC/FID can become useful for the CHNSO characterisation of volatile and semi-volatile organic liquid compounds in samples such as conventional fuels [33,34,35] and light oil fractions obtained from pyrolysis and liquefaction of biomass [36,37,38], plastics [39,40], coal [41,42], and their mixtures [43,44]. 

In this work, a combined GC/MS–GC/FID method was used to resolve the challenges of using conventional analysers for determining the CHNSO contents of volatile organic liquid products. The use of qualitative GC/MS and quantitative GC/FID techniques offers the potential for research and development of new automated systems for accurate simultaneous determinations of concentrations of individual components and elemental compositions of organic mixtures such as liquid fuels and other volatile liquid samples and feedstocks. This novel study could help to achieve better results for the elemental compositions consistent with the actual chemical compositions of oil products containing high proportions of volatile and semi-volatile organic compounds. 

## 2. Results

### 2.1. CHNSO Results of Simulated Hydrocarbon Oil Mixtures

The results from the two analytical methods and the “Theoretical” values, calculated from the molecular formulae of the compounds, are presented in Table 1. The results show that the CHNSO analyser produced unrealistic results. 

For instance, all the compounds used in the mixtures were hydrocarbons (made up of carbon and hydrogen atoms only), but the CHNS analyser (Method 1) reported high oxygen contents (by difference) in mixtures containing lower molecular weight (more volatile) hydrocarbons (Mixture 2 and Mixture 3). In addition to returning high oxygen values for Mixture 2 (16.48%) and Mixture 3 (24.33%), Table 1 also shows the large percentage standard deviations in the reported oxygen values of ±18% and ±7.10, respectively, from Method 1. This indicated that the standard CHNSO method using an elemental analyser with a pre-loaded autosampler was not appropriate for determining the elemental compositions of volatile liquid hydrocarbons. Indeed, the results showed that Mixture 2 containing more volatile compounds (o-xylene, toluene, and decane) resulted in a %SD value (±18 wt%) that was larger than the ‘actual’ oxygen content by difference (16.48 wt%). Hence, the volatile losses led to lower carbon and hydrogen contents, and the differences were erroneously reported as oxygen contents. Such errors are enhanced for volatile organic materials when obtaining “oxygen by difference” during standard CHNS analysis [45,46]. 

In contrast, the results from the GC/FID (Method 2) gave very good similarities with those theoretically calculated (theoretical) for all three mixtures. All the compounds in the three mixtures were completely identified by the GC/MS (Supplementary Materials Figures S1–S3) and quantified by the GC/FID. Hence, the GC/FID was able to produce representative elemental data for both the volatile and semi-volatile liquid hydrocarbons. These results demonstrated that with careful sample handling and accurate application, the combination of GC/MS and GC/FID can be a useful tool for accurate CHNSO analysis of volatile organic liquids. Interestingly, there were good matches between the elemental compositions of Mixture 1, which contained long-chain hydrocarbons, from all three methods. Hence, due to their low volatility, losses during waiting time for CHNS analyses were minimal, if at all. This confirmed the compatibility of solid organic material and non-volatile and semi-volatile organic liquids with the procedures of using the standard CHNS analyser [47,48].

### 2.2. Yields and Compositions of Oil-Pt/C, Oil-Pt/MgSiO_3_ and Oil-Pt/Al_2_O_3_

**Figure 1:**
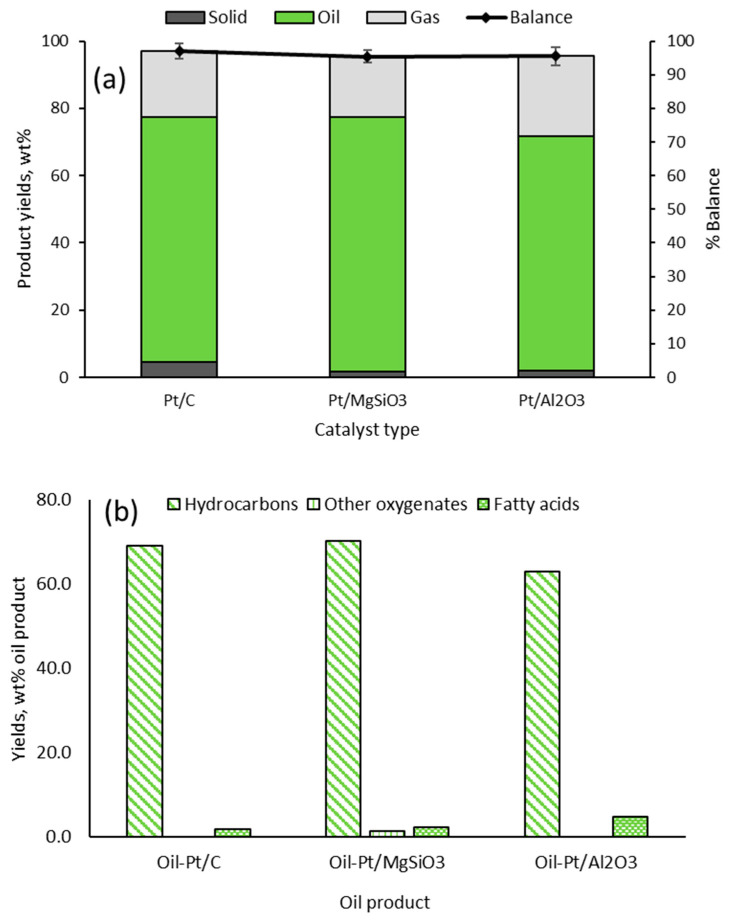
Results from catalytic deoxygenation of hydrolysed RSO; (**a**) product yields (**b**) compositions of the final organic liquid products (average values with SD < 2.5%).

Figure 1a presents yields of products from the catalytic deoxygenation experiments for conversion of hydrolysed RSO, with mass balance closures over 95%. The oil product was dominant, accounting for ≥70 wt% of reaction products, followed by gas products (from cracking). Char (solid) yields were minimal, with the Pt/C catalyst producing the highest yield of 4.7 wt%. However, with a focus on the accurate CHNSO determination of the oil products, detailed characterisations of the gas and solid products were deemed outside the scope of this present work.

The compositions of Oil-Pt/C, Oil-Pt/MgSiO_3_, and Oil-Pt/Al_2_O_3_ are shown in Figure 1b. Qualitative and quantitative analyses of hydrocarbons and ‘other oxygenates’ in the oils were determined by GC/MS and GC/FID (Section 4), whereas the fatty acids were determined as oleic acid by acid-base back titration. Hence, the combination of gas chromatography and back titration methods was able to accurately identify and quantify more than 95% of the components in the oil products. 

The high yields of hydrocarbons (>90 wt% on an oil product basis) confirmed that the combination of reaction conditions and the catalysts were effective in deoxygenating the fatty acids in the feedstock. Indeed, the catalysts were found to have promoted the simultaneous decarboxylation and cracking of the fatty acids, producing a wide range of hydrocarbons from hexane to nonadecane (Table 2, Table 3 and Table 4). A few oxygenated compounds, such as fatty alcohols and esters, were identified in *Oil-Pt*/*MgSiO_3_*, while all three samples showed the presence of unconverted fatty acids (reported here as oleic acid). The results showed that the Pt/Al_2_O_3_ catalyst was the least efficient in converting fatty acids, with 6.63 wt% remaining unconverted after the reaction. These oil products were used for CHNSO determination by conventional elemental analyser (Method 1) and by GC/FID (Method 2).

### 2.3. CHNSO Results of RSO-Derived Organic Liquid Products

Given the success of the GC/FID-based CHNSO characterisation of known volatile and non-volatile compounds in the prepared mixtures, the method was applied to the three samples of hydrocarbon-rich oil products obtained from hydrolysed RSO. Initial characteristics of the RSO showed that it was composed of 77.0 ± 1.02 wt% carbon, 11.0 ± 0.15 wt% hydrogen, 10.9 ± 1.42 wt% oxygen, no sulphur, and almost no nitrogen (0.13 ± 0.01 wt%) [49]. Indeed, no nitrogen-containing compound was detected during the qualitative GC/MS analyses of *Oil-Pt*/*C*, *Oil-Pt*/*MgSiO_3_*, and *Oil-Pt*/*Al_2_O_3_*. Hence, it could be concluded that the hydrocarbon-rich oil products obtained from RSO should only contain carbon, hydrogen, and oxygen (CHO) atoms (Supplementary Materials Figures S4–S6). The different calculations carried out to determine the CHO contents of the three RSO-derived organic liquid samples are presented in Table 2, Table 3 and Table 4. The Retention Indices (RI) of most of the compounds identified in the oils were calculated and included in the tables.

## 3. Discussion

**Figure 2:**
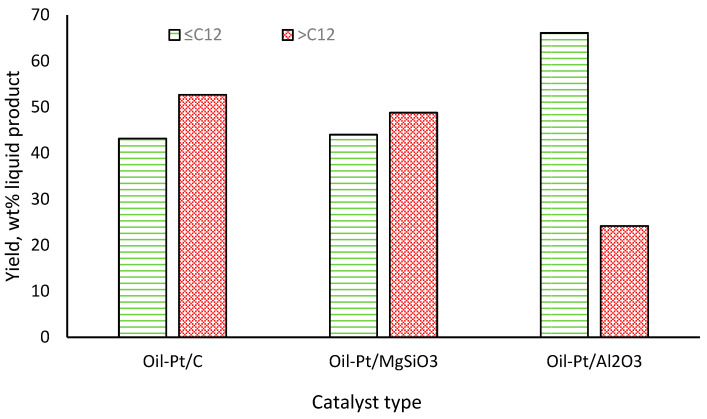
Distributions of hydrocarbons in the RSO-derived organic liquids based on carbon chain lengths (average values, with SD < 3%).

In all three oil products, heptadecane was the dominant hydrocarbon product, having been formed from the initial decarboxylation of oleic acid and other C_18_ fatty acids present in the RSO feedstock [49]. The catalysts performed differently in terms of heptadecane yield, with 5 wt% Pt/C and Pt/MgSiO_3_ catalysts producing 30.16 wt% and 27.66 wt% of the hydrocarbon, respectively, while their yield was only 11.7 wt% when Pt/Al_2_O_3_ was used as a catalyst. While the detailed activities of the catalysts are beyond the scope of this present work, it is important to note that the range of hydrocarbons in the oil products would influence the results of the CHNSO analysis using the elemental analyser. Figure 2 shows the distribution of the hydrocarbons in the three oil products into light volatile hydrocarbons (≤C_12_) and heavier semi-volatile hydrocarbons (>C_12_). This distribution shows that the oil product obtained in the presence of Pt/Al_2_O_3_ contained the highest yields of light volatile hydrocarbons (66.1 wt%), while the Pt/C catalyst gave the highest yields of heavier semi-volatile hydrocarbons (52.7 wt%).

From the results obtained using the simulated organic liquids (Section 2.1), it was clear that the presence of lighter hydrocarbons led to considerable losses during the conventional CHNSO analysis by Method 1, erroneously reporting the presence of “oxygen by difference” for pure hydrocarbon molecules. Hence, the presence and compositions of light volatile hydrocarbons in the RSO-derived oil products significantly affected the accuracy of the CHNSO analysis using the conventional elemental analyser. Table 5 shows the CHO data of all three RSO-derived organic liquid products obtained from both the conventional CHNSO analyser (Method 1) and the GC/FID (Method 2). The results showed that the GC/FID data consistently gave higher carbon and hydrogen contents and much less oxygen content compared to Method 1. The trend in the elemental data appeared to correspond to the compositions of the oils, with increased fatty acid conversion to hydrocarbons and increased volatility of the final oil products. For instance, the *Oil-Pt*/*C* would be the least volatile among the three oil products, considering the yields of >C_12_ hydrocarbons (52.7 wt%). (Figure 2). This could explain why, even though *Oil-Pt*/*MgSiO*_3_ contained similar yields of <C_12_ hydrocarbons as *Oil-Pt*/*C*, its lower >C_12_ content would make it more volatile than *Oil-Pt*/*C*. This observation may explain the observed differences in the ‘oxygen by difference’ values for these two samples using Method 1.

### Commentary and Future Perspectives

This present work has highlighted the need to understand the incompatibility of volatile organic liquids with traditional CHNSO analysers caused by inevitable sample losses during analysis. While there are potential solutions to achieving better accuracy with traditional analysers, the ones mentioned here are by no means exhaustive, but they have their peculiar challenges. Solutions could involve the following: (1) Analyser manufacturers could find means of eliminating volatile losses, e.g., by designing temperature-programmable sample holders that can operate at sufficiently low temperatures, but this would lead to additional procurement and potentially maintenance costs. (2) Manufacturers could ensure that instruments take account of volatile losses by recording sample masses over time and using such for processing CHNSO data. However, there would be the complications of changes in sample compositions following volatile losses. (3) Analysts could attempt to accurately time the loading of samples on to the analysers to minimise waiting time on sample holders, but again, this would involve loss of valuable users’ time while invalidating the principles of automation.

Clearly, the results from this present work have shown that elemental analysis of volatile liquids by qualitative and quantitative GC can provide more accurate results than traditional CHNSO analysers. However, it is important to also acknowledge the complications associated with the use of GC for this purpose in terms of equipment costs as well as the advanced levels of skills required of users for sample preparation, data analysis, interpretation, and validation. Essentially, GCs are much more expensive than traditional analysers, so it must be emphasised that the combined GC method would be recommended for liquid samples with significant issues around volatility losses and in situations when accurate CHNSO characterisation data are needed for further applications. Notwithstanding these challenges, in this growing era of artificial intelligence (AI) and machine learning applications, it may be possible to develop this combined GC/MS–GC/FID method along with appropriate data processing capability for automation. It may therefore be of interest to analytical equipment manufacturers to investigate how such a concept may be achieved at affordable costs. Hence, further research can lead to the development of such automated systems that could simultaneously provide accurate determinations of concentrations of individual components and elemental compositions of volatile organic mixtures such as liquid fuels. The alternative is to continue using traditional analysers to report grossly inaccurate CHNSO data for volatile liquid samples or completely ignore these types of data for such samples.

## 4. Materials and Methods

Three mixtures of hydrocarbons with high to medium volatilities were prepared as shown in Table 6. The compounds were selected based on their presence in organic liquid products obtained from the deoxygenation (catalytic decarboxylation) of fatty acids derived from RSO (Section 2.1).

Six samples of each mixture were prepared and three samples of each used for elemental analyses with a conventional Flash 2000 elemental analyser (Thermo Fisher Scientific, Cambridge, UK) (Method 1) using 2–3 mg of each sample. In the procedure, each of the samples was weighed out and immediately loaded onto the elemental analyser one at a time to keep waiting times before analysis the same. This was designed as a control measure to minimise volatility losses.

Compounds in the simulated and real samples were first identified using GC/MS, and then a GC/FID method (Method 2) was used to quantify each of the three samples. The GC/MS equipment (for identification) used was the Shimadzu GC-2010 GC/MS model, fitted to a Shimadzu MS-QP2010 SE (Shimadzu, Milton Keynes, UK). The GC/MS was used in the electron impact (EI) ionisation mode, scanning from a mass/charge (*m*/*z*) ratio of 35 to 500 during the analysis. The column used was an Rtx®-5MS (Restek, Ripley, UK), fused silica column with a 5% diphenyl/95% dimethyl polysiloxane phase(ID 0.25 mm, 30 m in length) with helium as carrier gas at a flowrate of 15 mL/min. Each sample shown in Table 1 was introduced into the injector held at 250 °C using a split ratio of 1:20. The oven programme was as follows: start at 40 °C and hold for 5 min, ramp at 8 °C/min to 185 °C, and then ramp at 14 °C/min to 280 °C and hold for 2 mins to give a total analysis time of 30.66 min. All the components in the simulated samples were correctly identified using the installed NIST library (NIST 2020, National Institute of Standards and Technology, Gaithersburg, MD, USA) prior to quantitation.

Quantitation of components in the known liquid mixtures (Table 6) was carried out by the internal standard method using a GC/FID (Shimadzu, GC-2010 Plus, Shimadzu, Milton Keynes, UK). The analysis was performed by using an Rtx^®^-5MS (Restek, Ripley, UK) fused silica column (with a 5% diphenyl/95% dimethyl polysiloxane phase, 30 m long, 0.25 mm id, 0.25 µm). The carrier gas (nitrogen) flow rate was set to 0.90 mL/min. Injector and detector temperatures were 300 °C and 280 °C, respectively. For analysis, 1 µL of each sample (simulated or real) was injected with a split ratio of 1:20. The same oven temperature programme for the GC/MS above was used. Diphenyl ether was used as the internal standard.

### 4.1. Preparation of the Hydrocarbon-Rich Liquid Samples

To further test the elemental analysis by the two methods, hydrocarbon-rich liquid samples were prepared from rapeseed oil (RSO) in a two-stage reaction. These oils were obtained as part of the extensive research into the conversion of RSO to fuel-range liquid hydrocarbons, and the details of the process are not within the scope of this paper. Briefly, the RSO was first quantitatively hydrolysed under hydrothermal conditions at 300 °C for 1 h to produce fatty acids as presented in a previous publication [49]. The fatty acids in the hydrolysed RSO were reacted to deoxygenate them by decarboxylation in the presence of three different platinum-based catalysts [49,50,51]. The catalysts, namely 5 wt% Pt/C, 5 wt% Pt/MgSiO_3_, and 5 wt% Pt/Al_2_O_3_, were reacted with hydrolysed RSO at 450 °C for 1 h of reaction time each to produce high yields of hydrocarbon-rich liquids (Table 2, Table 3 and Table 4 in Section 2.2). The decarboxylation tests were carried out in a 75 ml batch Hastelloy reactor [52]. In each case, the reactor was withdrawn from the heater once the set conditions were reached and placed in cold water to quench the reaction. The organic liquid products obtained were designated as *Oil-Pt*/*C*, *Oil-Pt*/*MgSiO_3_*, and *Oil-Pt*/*Al_2_O_3_* to indicate the type of catalyst used, respectively (Supplementary Materials Figure S7) and used for this present work.

### 4.2. Analysis of Fatty Acid Contents in Oil-Pt/C, Oil-Pt/MgSiO_3_, and Oil-Pt/Al_2_O_3_

Preliminary qualitative analysis of *Oil-Pt*/*MgSiO_3_* showed that the GC/MS identified mostly hydrocarbons (compounds containing carbon and hydrogen atoms only), and a few oxygenated organic compounds (esters and alcohols) in the oil. However, no fatty acids were detected, which could be due to the incompatibility of the type of GC column used for such compounds. Therefore, any unconverted fatty acids were determined in each oil product by a standard acid-base back titration based on a modified version of the Official AOCS:Cd-3a-63 (American Oil Chemists’ Society) method [53,54]. In the procedure, 4 mL of the sample of the oil product dissolved in dichloromethane (DCM) was mixed into 25 ml of a 0.1 M ethanolic solution of sodium hydroxide (NaOH). The mixture was back titrated with a standard 0.1 M HCl solution, using phenolphthalein as an indicator [49].

### 4.3. Analysis of Organic Liquid Products by Gas Chromatography

The same GC/MS and GC/FID and their temperature programmes used for the simulated hydrocarbon liquid mixtures were employed for the analysis of *Oil-Pt*/*C*, *Oil-Pt*/*MgSiO*_3_, and *Oil-Pt*/*Al_2_O_3_*. GC/MS was used for identification of the compounds in the oils, while GC/FID was used for their quantitation, again using diphenyl ether as the internal standard.

### 4.4. Evaluation of Experimental Results

Previous publication showed that oleic acid was the dominant fatty acid in the rapeseed oil, accounting for 74.4 wt% [49]. Therefore, the fatty acid contents of the oil products obtained after the catalytic decarboxylation tests were calculated based on the molecular weight of oleic acid according to Equation (1).

(1) $$\%\;Fatty\;acid\;yields==\frac{(B-S)\times N\times M}{10\times W}$$

where

*B* = volume of NaOH used in titration of blank (mL);

*S* = volume of NaOH used in titration of sample (mL);

*N* = concentration of NaOH used (mol/L);

*W* = weight of sample (g);

*M* = molecular mass of fatty acid (282.5 g/mol for oleic acid).

The elemental compositions of the three simulated hydrocarbon liquid mixtures, *Oil-Pt*/*C*, *Oil-Pt*/*MgSiO*_3_, and *Oil-Pt*/*Al*_2_*O*_3_ oil samples obtained via the GC/FID method were calculated using Equations (2)–(4).

(2) $$Carbon\;content,\;C\;wt\%=\sum \frac{M_{C,i\times Y_{i}}}{M_{Ri}}$$

(3) $$Hydrogen\;content,\;H\;wt\%=\sum \frac{M_{H,i\times Y_{i}}}{M_{Ri}}$$

(4) $$Oxygen\;content,\;O\;wt\%=\sum \frac{M_{O,i\times Y_{i}}}{M_{Ri}}$$

where

$M_{C,i\;}$ = number of carbon atoms in compound *i* × 12.011;

$M_{H,i\;}$ = number of hydrogen atoms in compound *i* × 1.008;

$M_{O,i\;}$ = number of oxygen atoms in compound *i* × 15.999;

$Y_{i\;}$ = Yield of compound *i* in a given mixture from GC/FID analysis;

$Y_{i\;}$ = Molecular mass of compound *i* in the given mixture for GC/FID analysis.

At the end of the two sets of analyses, the averages and standard deviation values were calculated and reported.

In addition, for the simulated hydrocarbon oil samples, the theoretical carbon and hydrogen contents of each mixture were calculated (theoretical). These were based on the molecular formula of each compound and their mass fractions in the mixtures according to Equations (5)–(8).

(5) $$Theoretical\;carbon\;content\;in\;a\;given\;mixture={\sum C\;wt\%\;}_{i\;}\times x_{i}$$

(6) $${C\;wt\%}_{i}=\frac{No.\;of\;carbon\;atoms\;in\;compound\;i\times 12.001\times 100\;}{Mr\;of\;compound\;i}$$

(7) $$Theoretical\;hydrogen\;content\;in\;a\;given\;mixture={\sum H\;wt\%\;}_{i\;}\times x_{i}$$

(8) $${H\;wt\%}_{i}=\frac{No.\;of\;hydrogen\;atoms\;in\;compound\;i\times 1.008\times 100\;}{Mr\;of\;compound\;i}$$

where $x_{i}$ = mass fraction of compound *i* in a given Mixture.

## Figures and Tables

**Table 1:** Comparison between elemental contents of simulated oil mixtures by CHNS analyser and GC/FID.

Elemental Composition						
Standards	Analysis	C (wt%)	H (wt%)	N (wt%)	S (wt%)	O (wt%)
Mixture 1	Method 1	84.02 ± 0.23	15.41 ± 0.02	0.20 ± 0.00	0	0.47 ± 0.22
	Method 2	86.05 ± 0.49	13.95 ± 0.21	0	0	0
	Theoretical	86.10	13.90	0	0	0
Mixture 2	Method 1	71.53 ± 14.76	11.89 ± 0.02	0.10 ± 0.02	0	16.48 ± 18.0
	Method 2	86.94 ± 0.80	13.06 ± 0.11	0	0	0
	Theoretical	88.79	11.21	0	0	0
Mixture 3	Method 1	64.20 ± 6.11	11.35 ± 1.02	0.11 ± 1.02	0	24.33 ± 7.10
	Method 2	85.73 ± 0.58	14.27± 0.15	0	0	0
	Theoretical	86.90	13.10	0	0	0

**Table 2:** Evaluation of average GC/FID analytical data for CHO contents of RSO-derived oil obtained at 450 °C with 5 wt% Pt/C for 1 h.

RT (min)	Compounds Present	Formula	RI	No. C Atoms	No. H Atoms	No. O Atoms	No. of C Atoms × 12.011	No. of H Atoms × 1.008	No. of O Atoms × 15.999	Mol. Wt.	wt% Yield in Final Oil	wt% C	wt% H	wt% O
2.757	Heptane	C_7_H_16_	700	7	16	0	84.077	16.128	0.00	100.205	5.145	4.317	0.828	0.000
3.855	Toluene	C_7_H_8_	762	7	8	0	84.077	8.064	0.00	92.141	0.460	0.420	0.040	0.000
4.536	Octane	C_8_H_18_	800	8	18	0	96.088	18.144	0.00	114.232	6.276	5.279	0.997	0.000
6.463	Ethylbenzene	C_8_H_10_	863	8	10	0	96.088	10.08	0.00	106.168	0.665	0.602	0.063	0.000
7.416	o-Xylene	C_8_H_10_	895	8	10	0	96.088	10.08	0.00	106.168	1.357	1.228	0.129	0.000
7.581	Nonane	C_9_H_20_	900	9	20	0	108.099	20.16	0.00	128.259	6.572	5.539	1.033	0.000
9.208	Propyl benzene	C_9_H_12_	957	9	12	0	108.099	12.096	0.00	120.195	0.248	0.223	0.025	0.000
9.935	1-Ethyl-2-methylbenzene	C_9_H_12_	983	9	12	0	108.099	12.096	0.00	120.195	1.509	1.358	0.152	0.000
10.429	Decane	C_10_H_22_	1000	10	22	0	120.11	22.176	0.00	142.286	6.082	5.134	0.948	0.000
12.063	1,3-Diethylbenzene	C_10_H_14_	1066	10	14	0	120.11	14.112	0.00	134.222	0.450	0.403	0.047	0.000
12.194	1-Methyl-2-propylbenzene	C_10_H_14_	1071	10	14	0	120.11	14.112	0.00	134.222	0.961	0.860	0.101	0.000
12.689	1-Ethenyl-4-ethylbenzene	C_10_H_10_	1091	10	10	0	120.11	10.08	0.00	130.19	0.244	0.225	0.019	0.000
12.905	Undecane	C_11_H_24_	1200	11	24	0	132.121	24.192	0.00	156.313	5.633	4.761	0.872	0.000
14.013	(1,1-Dimethylpropyl) benzene	C_11_H_16_	1245	11	16	0	132.121	16.128	0.00	148.249	0.393	0.351	0.043	0.000
14.155	1-Phenyl-1-butene	C_10_H_12_	1150	10	12	0	120.11	12.096	0.00	132.206	0.322	0.293	0.029	0.000
14.489	1-Methyl-4-butylbenzene	C_11_H_16_	1264	11	16	0	132.121	16.128	0.00	148.249	0.555	0.494	0.060	0.000
14.938	Naphthalene	C_10_H_8_	1182	10	8	0	120.11	8.064	0.00	128.174	0.390	0.366	0.025	0.000
15.082	Dodecane	C_12_H_26_	1200	12	26	0	144.132	26.208	0.00	170.34	5.225	4.421	0.804	0.000
16.534	(1,3-dimethylbutyl) benzene	C_12_H_18_	1274	12	18	0	144.132	18.144	0.00	162.276	0.364	0.324	0.041	0.000
17.048	Tridecane	C_13_H_28_	1300	13	28	0	156.143	28.224	0.00	184.367	4.499	3.810	0.689	0.000
17.174	1-Methyl naphthalene	C_11_H_10_	1372	11	10	0	132.121	10.08	0.00	142.201	0.309	0.287	0.022	0.000
18.437	1-Methyl-2-n-hexylbenzene	C_13_H_20_	1377	13	20	0	156.143	20.16	0.00	176.303	0.305	0.270	0.035	0.000
18.858	Tetradecane	C_14_H_30_	1400	14	30	0	168.154	30.24	0.00	198.394	3.923	3.325	0.598	0.000
20.222	(1-Methylheptyl) benzene	C_14_H_22_	1481	14	22	0	168.154	22.176	0.00	190.33	0.296	0.262	0.035	0.000
20.547	Pentadecane	C_15_H_32_	1500	15	32	0	180.165	32.256	0.00	212.421	6.065	5.144	0.921	0.000
22.13	Hexadecane	C_16_H_34_	1600	16	34	0	192.176	34.272	0.00	226.448	1.709	1.451	0.259	0.000
22.777	2,6,10-Trimethyltridecane	C_16_H_34_	1647	16	34	0	192.176	34.272	0.00	226.448	0.763	0.648	0.116	0.000
22.949	4-Methyl pentadecane	C_16_H_34_	1660	16	34	0	192.176	34.272	0.00	226.448	0.467	0.397	0.071	0.000
23.018	2-Methyl hexadecane	C_17_H_36_	1657	17	36	0	204.187	36.288	0.00	240.475	1.135	0.964	0.171	0.000
23.125	3-Methyl hexadecane	C_17_H_36_	1667	17	38	0	204.187	38.304	0.00	242.491	0.813	0.685	0.128	0.000
23.506	Heptadecane	C_17_H_36_	1700	17	36	0	204.187	36.288	0.00	240.475	30.155	25.605	4.550	0.000
23.635	(1-Methyldecyl) benzene	C_17_H_28_	1711	17	28	0	204.187	28.224	0.00	232.411	0.674	2.173	0.300	0.000
24.222	(1,1-Dimethylnonyl) benzene	C_17_H_28_	1763	17	28	0	204.187	28.224	0.00	232.411	0.370	0.325	0.045	0.000
24.542	Undecyl benzene	C_17_H_28_	1790	17	28	0	204.187	28.224	0.00	232.411	0.346	0.304	0.042	0.000
24.651	Octadecane	C_18_H_38_	1800	18	38	0	216.198	38.304	0.00	254.502	0.500	0.425	0.075	0.000
25.663	Nonadecane	C_19_H_40_	1900	19	40	0	228.209	40.32	0.00	268.529	0.628	0.534	0.094	0.000
	Unreacted fatty acids (as Octadec-9-enoic acid)	C_18_H_36_O_2_	-	18	36	2	216.198	36.288	31.998	284.484	2.340	1.778	0.298	0.263
	Total for each element											84.983	14.705	0.263

RT = retention time; RI = Retention Index.

**Table 3:** Evaluation of average GC/FID analytical data for CHO contents of RSO-derived oil obtained at 450 °C with 5 wt% Pt/MgSiO_3_ for 1 h.

RT (min)	Compounds Present	Formula	RI	No. C Atoms	No. H Atoms	No. O Atoms	No. of C Atoms × 12.011	No. of H Atoms × 1.008	No. of O Atoms × 15.999	Mol. Wt.	wt% Yield in Final Oil	wt% C	wt% H	wt% O
2.022	Hexane	C_6_H_14_	600	6	14	0	72.066	14.112	0.000	86.178	2.270	1.898	0.372	0.000
2.462	2-Methyl hexane	C_7_H_16_	683	7	16	0	84.077	16.128	0.000	100.205	0.481	0.403	0.077	0.000
2.69	Hept-1-ene	C_7_H_14_	695	7	14	0	84.077	14.112	0.000	98.189	0.375	0.321	0.054	0.000
2.776	Heptane	C_7_H_16_	700	7	16	0	84.077	16.128	0.000	100.205	4.653	3.904	0.749	0.000
2.864	(E)-Hept-2-ene	C_7_H_14_	705	7	14	0	84.077	14.112	0.000	98.189	0.425	0.364	0.061	0.000
3.879	Toluene	C_7_H_8_	760	7	8	0	84.077	8.064	0.000	92.141	0.403	0.368	0.035	0.000
3.934	3-Methyl heptane	C_8_H_18_	776	8	18	0	96.088	18.144	0.000	114.232	0.344	0.289	0.055	0.000
4.349	Oct-1-ene	C_8_H_16_	791	8	18	0	96.088	18.144	0.000	114.232	0.252	0.212	0.040	0.000
4.563	2,4-Dimethy heptane	C_9_H_20_	793	9	20	0	108.099	20.16	0.000	128.259	5.733	4.831	0.901	0.000
4.757	(E)-Oct-2-ene	C_8_H_16_	805	8	16	0	96.088	16.128	0.000	112.216	0.240	0.205	0.034	0.000
6.489	Ethylbenzene	C_8_H_10_	863	8	10	0	96.088	10.08	0.000	106.168	0.542	0.491	0.051	0.000
6.732	(3,3-dimethylbutyl)-benzene	C_12_H_18_	-	12	18	0	144.132	18.144	0.000	162.276	0.358	0.318	0.040	0.000
7.351	Non-1-ene	C_9_HJ_18_	891	9	18	0	108.099	18.144	0.000	126.243	0.282	0.241	0.041	0.000
7.442	o-Xylene	C_8_H_10_	895	8	10	0	96.088	10.08	0.000	106.168	0.292	0.264	0.028	0.000
7.604	Nonane	C_9_H_20_	900	9	20	0	108.099	20.16	0.000	128.259	6.460	5.445	1.015	0.000
7.799	(E)-2-Nonene	C_9_H_18_	907	9	18	0	108.099	18.144	0.000	126.243	0.375	0.321	0.054	0.000
9.228	Propyl benzene	C_9_H_12_	957	9	12	0	108.099	12.096	0.000	120.195	0.156	0.140	0.016	0.000
9.661	3-Methyl nonane	C_10_H_22_	968	10	22	0	120.11	22.176	0.000	142.286	0.238	0.201	0.037	0.000
9.956	1-ethyl-2-methylbenzene	C_9_H_12_	983	9	12	0	108.099	12.096	0.000	120.195	0.261	0.235	0.026	0.000
10.324	1,3,5-trimethylbenzene	C_9_H_12_	996	9	12	0	108.099	12.096	0.000	120.195	0.259	0.233	0.026	0.000
10.45	Decane	C_10_H_22_	1000	10	22	0	120.11	22.176	0.000	142.286	6.412	5.412	0.999	0.000
10.607	(E)-Dec-2-ene	C_10_H20	1004	10	20	0	120.11	20.16	0.000	140.27	0.263	0.226	0.038	0.000
11.917	Ethyl 2-(4-isobutylphenyl)propionate	C_15_H_22_O_2_	-	15	22	2	180.165	22.176	31.998	234.339	0.951	0.716	0.090	0.147
12.077	2-Methyl decane	C_11_H_24_	1061	11	24	0	132.121	24.192	0.000	156.313	0.446	0.779	0.120	0.000
12.227	(1,3,3-Trimethylnonyl) benzene	C_18_H_30_	-	18	30	0	216.198	30.24	0.000	246.438	0.291	0.255	0.036	0.000
12.729	Octadecyl 2-ethylhexanoate	C_24_H_48_O	-	24	48	1	288.264	48.384	15.999	352.647	0.453	0.370	0.062	0.021
12.928	Undecane	C_11_H_24_	1100	11	24	0	132.121	24.192	0.000	156.313	5.883	4.973	0.911	0.000
14.041	(1,1-Dimethylpropyl) benzene	C_11_H_16_	1151	11	16	0	132.121	16.128	0.000	148.249	0.206	0.183	0.022	0.000
14.289	Pentyl benzene	C_11_H_16_	1163	11	16	0	132.121	16.128	0.000	148.249	0.161	0.143	0.018	0.000
14.49	3-Methyl undecane	C_12_H_26_	1169	12	26	0	144.132	26.208	0.000	170.34	0.254	0.215	0.039	0.000
15.105	Dodecane	C_12_H_26_	1200	12	26	0	144.132	26.208	0.000	170.34	5.455	4.616	0.839	0.000
16.29	4-Methyl dodecane	C_13_H_28_	1264	13	28	0	156.143	28.224	0.000	184.367	0.320	0.271	0.049	0.000
17.072	Tridecane	C_13_H_28_	1300	13	28	0	156.143	28.224	0.000	184.367	4.240	3.591	0.649	0.000
17.194	2-Methyl naphthalene	C_11_H_10_	1312	11	10	0	132.121	10.08	0.000	142.201	0.163	0.152	0.012	0.000
17.986	2-Hexyl decan-1-ol	C_16_H_34_O	1337	16	34	1	192.176	34.272	15.999	242.447	0.306	0.242	0.043	0.020
18.23	2-Methyl tridecane	C_14_H_30_	1361	14	30	0	168.154	30.24	0.000	198.394	0.231	0.196	0.035	0.000
18.883	Tetradecane	C_14_H_30_	1400	14	30	0	168.154	30.24	0.000	198.394	3.506	2.972	0.534	0.000
20.575	Pentadecane	C_15_H_32_	1500	15	32	0	180.165	32.256	0.000	212.421	5.872	4.980	0.892	0.000
22.159	Hexadecane	C_16_H_34_	1600	16	34	0	192.176	34.272	0.000	226.448	1.645	1.396	0.490	0.000
22.804	2,6,10-Trimethyltridecane	C_16_H_34_	1641	16	34	0	192.176	34.272	0.000	226.448	2.266	1.923	0.343	0.000
22.895	5-Methyl tetradecane	C_15_H_32_	1646	15	32	0	180.165	32.256	0.000	212.421	0.582	0.494	0.088	0.000
23.153	2-Methyl heptadecane	C_18_H_38_	1649	18	38	0	216.198	38.304	0.000	254.502	0.450	0.383	0.068	0.000
23.538	Heptadecane	C_17_H_36_	1700	17	36	0	204.187	36.288	0.000	240.475	27.660	23.486	4.174	0.000
24.68	Octadecane	C_18_H_38_	1800	18	38	0	216.198	38.304	0.000	254.502	0.428	0.363	0.064	0.000
25.693	Nonadecane	C_19_H_40_	1900	19	40	0	228.209	40.32	0.000	268.529	0.435	0.370	0.065	0.000
	Unreacted fatty acids (as Octadec-9-enoic acid)	C_18_H_36_O_2_	-	18	36	2	216.198	36.288	31.998	284.484	2.980	2.265	0.380	0.335
	Total for each element											81.660	14.774	0.523

RT = retention time; RI = Retention Index.

**Table 4:** Evaluation of average GC/FID analytical data for CHO contents of RSO-derived oil obtained at 450 °C with 5 wt% Pt/Al_2_O_3_ for 1 h.

RT (min)	Compounds Present	Formula	RI	No. C Atoms	No. H Atoms	No. O Atoms	No. of C Atoms × 12.011	No. of H Atoms × 1.008	No. of O Atoms × 15.999	Mol. Wt.	wt% Yield in Final Oil	wt% C	wt% H	wt% O
2.021	Hexane	C_6_H_14_	600	6	14	0	72.066	14.112	0.000	86.178	4.065	3.399	0.666	0.000
2.207	Methyl cyclopentane	C_6_H_12_	625	6	12	0	72.066	12.096	0.000	84.162	0.619	0.530	0.089	0.000
2.468	Benzene	C_6_H_6_	659	6	6	0	72.066	6.048	0.000	78.114	1.034	0.954	0.080	0.000
2.694	1,2-Dimethyl cyclopentane	C_7_H_14_	695	7	14	0	84.077	14.112	0.000	98.189	0.430	0.368	0.062	0.000
2.777	Heptane	C_7_H_16_	700	7	16	0	84.077	16.128	0.000	100.205	6.819	5.721	1.098	0.000
3.12	Methyl cyclohexane	C_7_H_14_	719	7	14	0	84.077	14.112	0.000	98.189	0.407	0.349	0.059	0.000
3.276	Ethyl cyclopentane	C_7_H_14_	727	7	14	0	84.077	14.112	0.000	98.189	0.599	0.513	0.086	0.000
3.878	Toluene	C_7_H_8_	760	7	8	0	84.077	8.064	0.000	92.141	2.378	2.170	0.208	0.000
4.565	2,4-Dimethyl heptane	C_9_H_20_	793	9	20	0	108.099	20.160	0.000	128.259	7.509	6.329	1.180	0.000
5.552	Propyl cyclopentane	C_8_H_16_	832	8	16	0	96.088	16.128	0.000	112.216	0.354	0.304	0.051	0.000
6.488	Ethylbenzene	C_7_H_10_	863	8	10	0	96.088	10.080	0.000	106.168	1.279	1.157	0.121	0.000
6.731	p-Xylene	C_7_H_10_	871	8	10	0	96.088	10.080	0.000	106.168	0.823	0.745	0.078	0.000
7.442	o-Xylene	C_7_H_10_	895	8	10	0	96.088	10.080	0.000	106.168	1.584	1.433	0.150	0.000
7.606	Nonane	C_9_H_20_	900	9	20	0	108.099	20.160	0.000	128.259	7.299	6.152	1.147	0.000
7.813	trans-1,2-Diethyl cyclopentane	C_9_H_18_	907	9	18	0	108.099	18.144	0.000	126.243	0.433	0.371	0.062	0.000
9.229	Propyl benzene	C_9_H_12_	957	9	12	0	108.099	12.096	0.000	120.195	0.495	0.445	0.050	0.000
9.455	1-Ethyl-3-methyl benzene	C_9_H_12_	965	9	12	0	108.099	12.096	0.000	120.195	2.065	3.428	0.384	0.000
10.329	1,3,5-trimethylbenzene	C_9_H_12_	996	9	12	0	108.099	12.096	0.000	120.195	0.966	0.869	0.097	0.000
10.451	Decane	C_10_H_22_	1000	10	22	0	120.110	22.176	0.000	142.286	6.764	5.709	1.054	0.000
11.453	Indane	C_9_H_10_	1035	9	10	0	108.099	10.080	0.000	118.179	0.552	0.505	0.047	0.000
11.941	n-Butyl benzene	C_10_H_14_	1060	10	14	0	120.110	14.112	0.000	134.222	0.659	0.590	0.069	0.000
12.084	1,3-Diethyl benzene,	C_10_H_14_	1066	10	14	0	120.110	14.112	0.000	134.222	0.321	0.287	0.034	0.000
12.216	1-Methyl-2-propyl benzene	C_10_H_14_	1071	10	14	0	120.110	14.112	0.000	134.222	0.595	0.533	0.063	0.000
12.505	1-Methyl-3-(1-methylethyl) benzene	C_10_H_14_	1083	10	14	0	120.110	14.112	0.000	134.222	0.193	0.173	0.020	0.000
12.71	1-Methyl indane	C_10_H_12_	1091	10	12	0	120.110	12.096	0.000	132.206	0.398	0.362	0.036	0.000
12.927	Undecane	C_11_H_24_	1100	11	24	0	132.121	24.192	0.000	156.313	5.814	4.914	0.900	0.000
13.166	1-methyl-4-isopropylbenzene	C_10_H_14_	1110	10	14	0	120.110	14.112	0.000	134.222	0.184	0.165	0.019	0.000
13.927	2,3-Dihydro-5-methyl-1H-indene	C_10_H_12_	1146	11	14	0	132.121	14.112	0.000	146.233	0.287	0.259	0.028	0.000
14.037	(1,1-Dimethylpropyl) benzene	C_11_H_16_	1151	11	16	0	132.121	16.128	0.000	148.249	0.401	0.358	0.044	0.000
14.177	1-Methyl-2-(2-propenyl) benzene	C_10_H_12_	1150	10	12	0	120.110	12.096	0.000	132.206	0.685	0.623	0.063	0.000
14.291	Pentyl benzene	C_11_H_16_	1163	11	16	0	132.121	16.128	0.000	148.249	0.466	0.415	0.051	0.000
14.511	1-Methyl-4-butyl benzene	C_11_H_16_	1173	11	16	0	132.121	16.128	0.000	148.249	0.335	0.299	0.036	0.000
14.954	Naphthalene	C_10_H_8_	1182	10	8	0	120.110	8.064	0.000	128.174	1.060	0.993	0.067	0.000
15.103	Dodecane	C_12_H_26_	1200	12	26	0	144.132	26.208	0.000	170.340	5.523	4.674	0.850	0.000
15.209	2,3-Dihydro-1,6-dimethyl-1H-indene	C_11_H_14_	1205	11	14	0	132.121	14.112	0.000	146.233	0.368	0.333	0.036	0.000
16.395	Hexyl benzene	C_12_H_18_	1266	12	18	0	144.132	18.144	0.000	162.276	0.383	0.340	0.043	0.000
16.555	(1,3-Dimethylbutyl) benzene	C_10_H_14_	1247	10	14	0	120.110	14.112	0.000	134.222	0.348	0.311	0.037	0.000
17.069	Tridecane	C_13_H_26_	1300	13	26	0	156.143	26.208	0.000	182.351	3.973	3.402	0.571	0.000
17.533	2- Methyl naphthalene	C_11_H_10_	1312	11	10	0	132.121	10.080	0.000	142.201	1.188	1.104	0.084	0.000
18.881	Tetradecane	C_14_H_30_	1400	14	30	0	168.154	30.240	0.000	198.394	2.935	2.488	0.447	0.000
19.034	2-Ethyl naphthalene	C_12_H_12_	1400	12	12	0	144.132	12.096	0.000	156.228	0.220	0.203	0.017	0.000
19.496	1,4-Dimethyl naphthalene	C_12_H_12_	1423	12	12	0	144.132	12.096	0.000	156.228	0.202	0.186	0.016	0.000
20.571	Pentadecane	C_15_H_32_	1500	15	32	0	180.165	32.256	0.000	212.421	4.207	3.568	0.639	0.000
22.157	Hexadecane	C_16_H_34_	1600	16	34	0	192.176	34.272	0.000	226.448	1.379	1.171	0.209	0.000
23.519	Heptadecane	C_17_H_34_	1700	17	34	0	204.187	34.272	0.000	238.459	11.696	10.015	1.681	0.000
	Unreacted fatty acids (as Octadec-9-enoic acid)	C_18_H_36_O_2_	-	18	36	2	216.198	36.288	31.998	284.484	6.625	5.035	0.845	0.745
	Total for each element											84.248	13.672	0.745

RT = retention time; RI = Retention Index.

**Table 5:** Results of CHO analysis of RSO-derived organic liquid products using the two methods.

Elemental Compositions						
Sample Code	Analysis	C (wt%)	H (wt%)	N (wt%)	S (wt%)	O (wt%)
Oil-Pt/C	Method 1	83.31 ± 3.20	13.93 ± 0.51	0.20 ± 0.00	nd	2.56 ± 0.08
	Method 2	84.99 ± 0.22	14.70 ± 0.53	nd	nd	0.26 ± 0.05
Oil-Pt/MgSiO_3_	Method 1	68.58 ± 0.52	11.80 ± 0.16	0.13 ± 0.02	nd	19.49 ± 0.13
	Method 2	82.28 ± 0.27	14.27 ± 0.08	nd	nd	0.52 ± 0.10
Oil-Pt/Al_2_O_3_	Method 1	59.24 ± 0.62	9.04 ± 0.08	0.13 ± 0.01	nd	31.59 ± 0.14
	Method 2	84.20 ± 0.88	13.72± 0.46	nd	nd	0.75 ± 0.12

nd = not detected.

**Table 6:** Solvent-free mixtures of hydrocarbons for elemental analysis by a CHNS analyser and the GC/FID method (0.1 g of each compound used in each mixture).

Heavy Hydrocarbons (Mixture 1)	Light Hydrocarbons (Mixture 2)	Combined Light and Heavy Hydrocarbons (Mixture 3)
Hexadecane Heptadecane Octadecane	Ortho-xylene Toluene Decane	Ortho-xylene Toluene Decane Hexadecane Heptadecane Octadecane

## Data Availability

All relevant data generated from this research are presented in the main manuscript and Supplementary Materials.

## Supplementary Materials

The following supporting information can be downloaded at: https://www.mdpi.com/article/10.3390/molecules29184346/s1, Figure S1: GC/MS chromatogram of hydrocarbons in Mixture 1 (diphenyl ether as internal standard); Figure S2: GC/MS chromatogram of hydrocarbons in Mixture 2 (diphenyl ether as internal standard); Figure S3: GC/MS chromatogram of hydrocarbons in Mixture 3 (diphenyl ether as internal standard); Figure S4: GC/MS chromatogram of Oil-Pt/C (diphenyl ether as internal standard); Figure S5: GC/MS chromatogram of Oil-Pt/MgSiO_3_ (diphenyl ether as internal standard); Figure S6: GC/MS chromatogram of Oil-Pt/Al_2_O_3_ (diphenyl ether as internal standard; Figure S7: Photos of the final organic liquid products obtained from catalytic deoxygenation of RSO.
