# Supplementary material for: CHNSO Elemental Analyses of Volatile Organic Liquids by Combined GC/MS and GC/Flame Ionisation Detection Techniques with Application to Hydrocarbon-Rich Biofuels
Source: Molecules. 2024 Sep 13;29(18):4346. doi: 10.3390/molecules29184346 (PMC11434348; doi:10.3390/molecules29184346)
Supplement: Supplementary file 1 [file molecules-29-04346-s001.zip › molecules-3190551-supplementary.pdf]

Supplementary Information: CHNSO Elemental Analyses of Volatile Organic Liquids by Combined GC/MS and GC/FID Techniques with Application to Hydrocarbon-rich Biofuels

Jude A. Onwudili[1, *], Morenike, A. Peters[1], Carine Tondo Alves[1,2],

[1]*Energy and Bioproducts Research Institute, College of Engineering and Physical Sciences, Aston University, Aston Triangle, Birmingham B4 7ET, UK*

[2]*Energy Engineering Department, Universidade Federal do Reconcavo da Bahia, CETENS, Av. Centenario 697, Feira de Santana, 44.085-132*

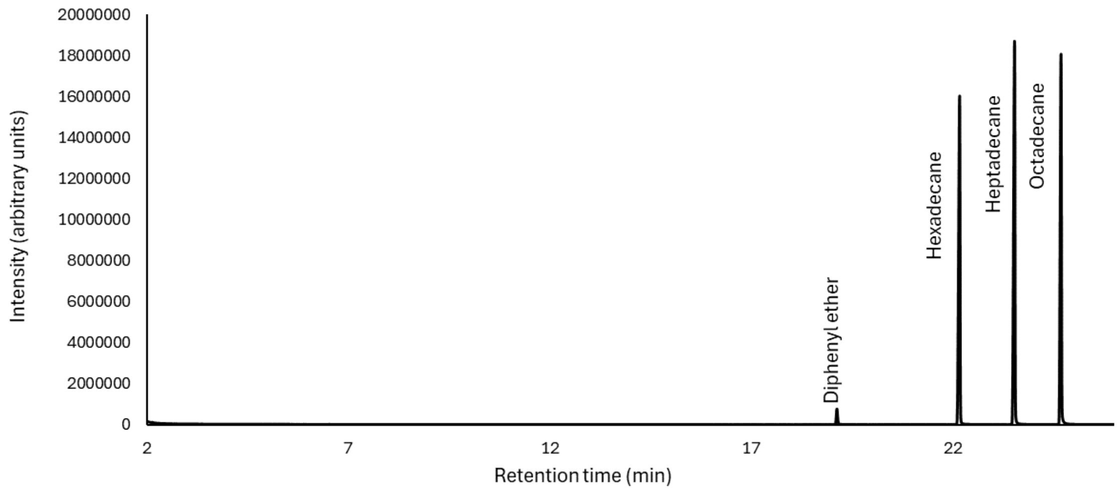

Figure S1: GC/MS chromatogram of hydrocarbons in Mixture 1 (diphenyl ether as internal standard)

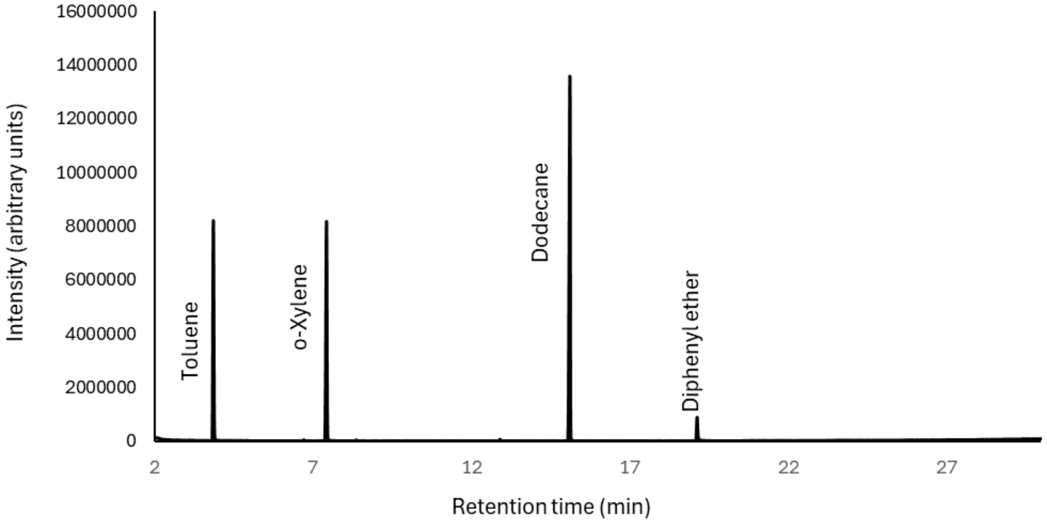

Figure S2: GC/MS chromatogram of hydrocarbons in Mixture 2 (diphenyl ether as internal standard)

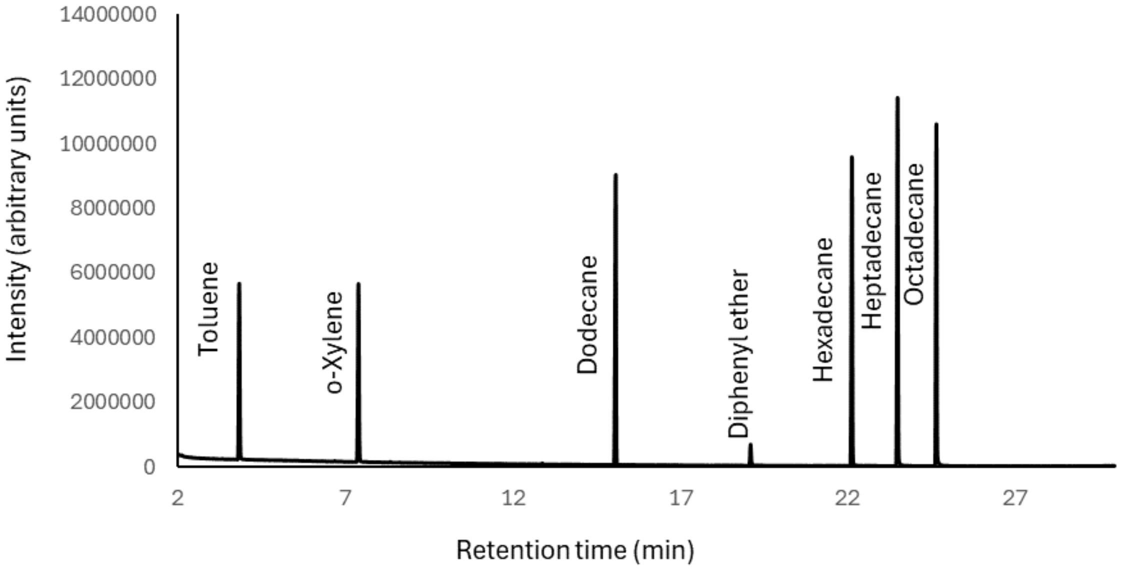

Figure S3: GC/MS chromatogram of hydrocarbons in Mixture 3 (diphenyl ether as internal standard)

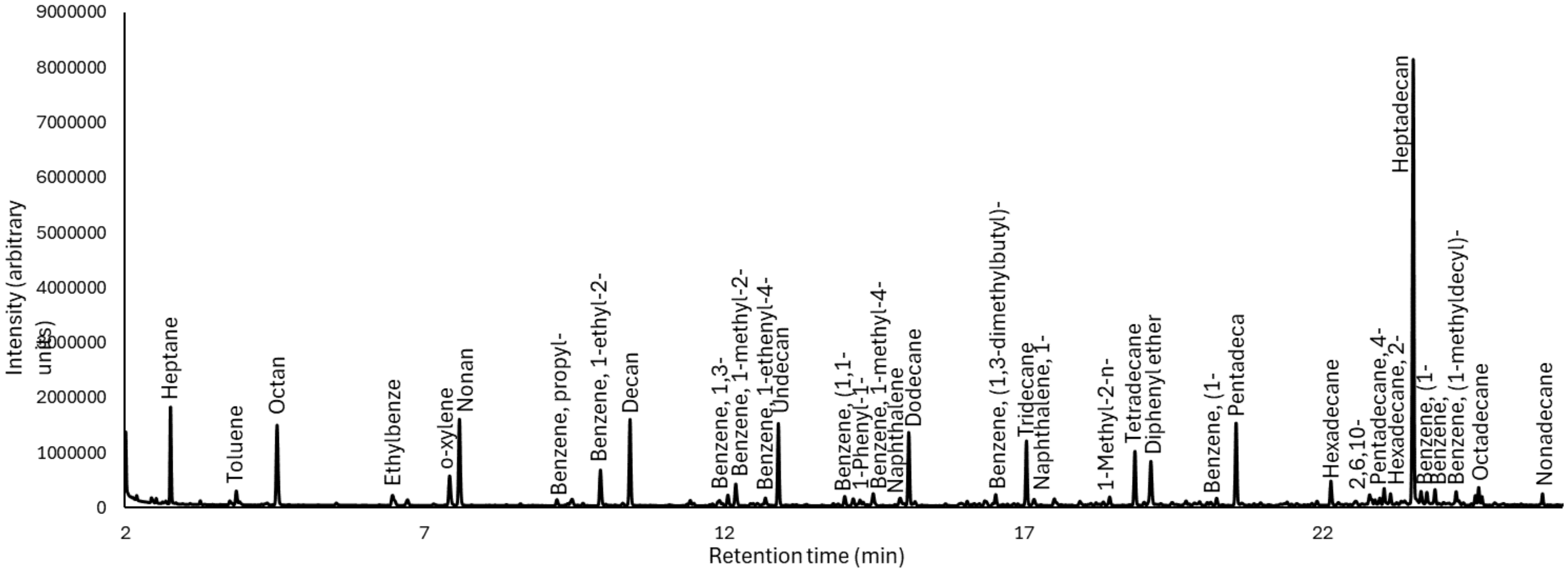

Figure S4: GC/MS chromatogram of Oil-Pt/C (diphenyl ether as internal standard)

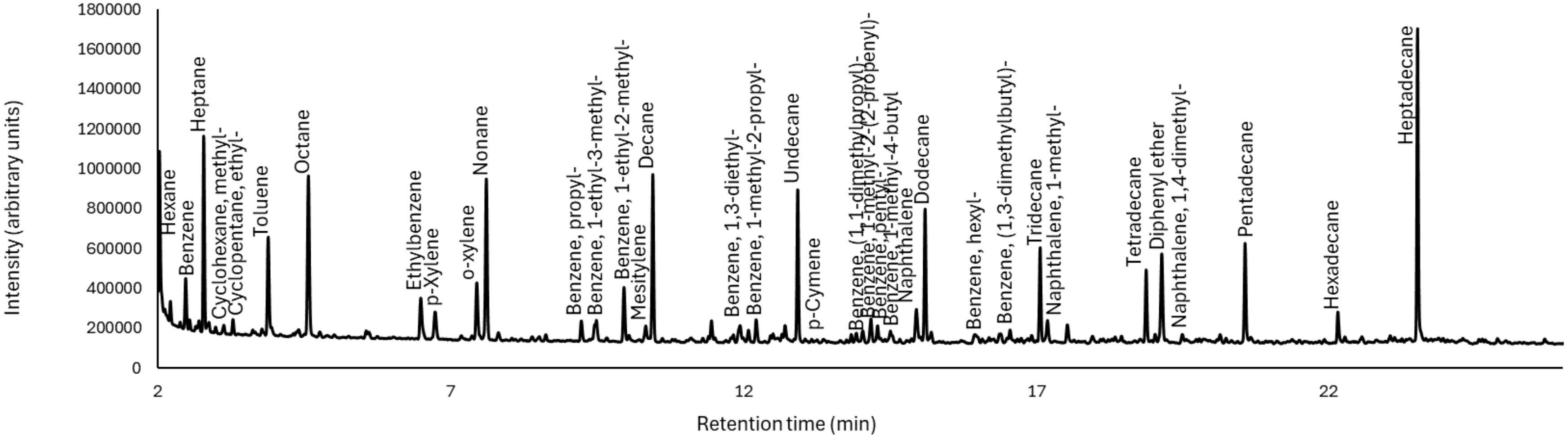

Figure S5: GC/MS chromatogram of Oil-Pt/$MgSiO_3$ (diphenyl ether as internal standard)

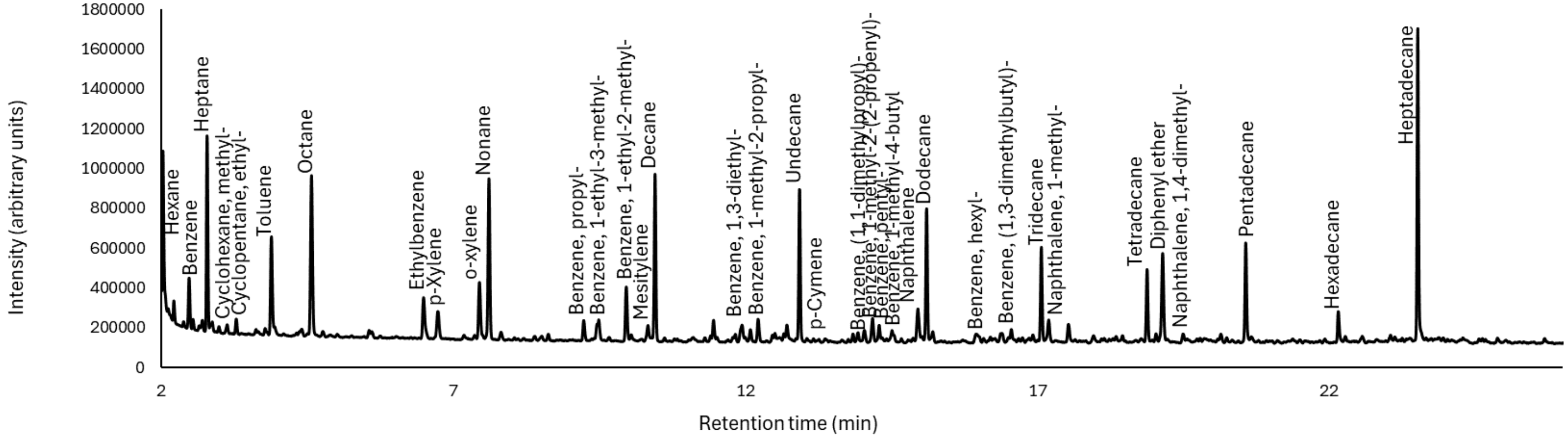

Figure S6: GC/MS chromatogram of Oil-Pt/Al_2O_3 (diphenyl ether as internal standard)

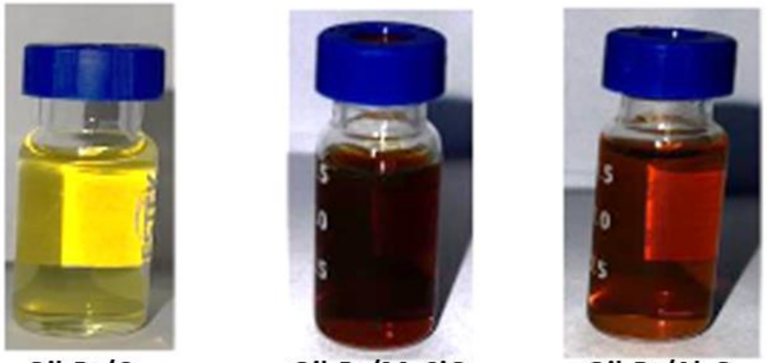

Oil-Pt/C   Oil-Pt/$MgSiO_3$ Oil-Pt/Al_2O_3

Figure S7: Photos of the final organic liquid products obtained from catalytic deoxygenation of RSO
